# Mississippi Valley Association of Dental Surgeons

**Published:** 1858-07

**Authors:** 


### ARTICLE VIII.

*Mississippi Valley Association of Dental Surgeons.* Fourteenth Annual Meeting.

WE had the pleasure of attending this meeting, now the "oldest dental society," last February, and met with a very cordial reception from old friends. That society, and the spot where its meetings are held, are centres, around which clusters much of our professional associations. We hope it may long continue to enjoy the right of age, and that to this it may add wisdom.

Although unable to attend closely all its sessions, when present we noted a few thoughts, some of which were not caught by Dr. Watt, who prepared the excellent report published in the Register.

### *Discussion on Inflamed Dentine.*

Dr. Taylor believed one-half of the disease we had to treat originated by want of care in the mother, after birth. Before birth, however, care must be given to the condition of things to secure good teeth. Believed it the duty of mothers, in many cases, to give their children to more healthy nurses, to insure health and healthy teeth.

Dr. Taft.—To secure healthy dentine, care must begin before birth. All undue excitement of the mind and peevishness of the mother must be avoided. Bad teeth, or lack of bone material in the mother, are imparted generally to the child. The latter is shown by presence of large fontanel on head of the child. Has administered phos. lime to some patients when this tendency was known to exist, and observed marked improvement.

Dr. Blandy.—What can we do to produce healthy dentine? But little, medicinally. We can only be guided by those general rules of hygiene which tend to promote health. Useless to decide to give those substances which will furnish most bone material, as that may not suit the case, objects to feeding before the grinding teeth are furnished, say end of second year. Cleanliness, even before the teeth are cut, essential, he thought, to secure sound teeth. Holds to filing and filling the temporary teeth.

Dr. Watt.—The physical and chemical composition of the child's teeth are derived more from the mother than the father. If dental materials are deficient in mother, would administer those which are calculated to build up. Life is but the result of chemical action of the nourishment taken into the system. If the gelatinous portion is deficient, would prescribe exercise and air. If chemical constituents were wanting, would analyze the milk. and if phosphate of lime was deficient, would administer it. Have seen a decided advantage from its use. If lactic acid is present, you will find abrasion of the surface of the teeth. When decay has lost its earthy portion, it shows the action of hydrochloric acid, would order diminution of seasoning in the food. When white decay exists and tooth entirely broken down, the cause is nitric acid, or nitrogenous substances.

In reply to query, Dr. Watt said, there was no acid in the stomach strong enough to decompose phosphate of lime.

Dr. Blakesly suggested that Dr. Watt write a book on the subject, and we would buy it and read it.

### *Discussion on "What is most effectual Treatment of Exposed Nerves?"*

Dr. Taft.—If nerve is exposed at a small point, cuts carefully, lays in oil silk, gutta percha, or two or three folds of foil, with asbestos between, attaches in position with adhesive gum. Leaves no space, finds no harm by pressure in such cases. If inflammation exists, tries local treatment to reduce it, succeeds in four cases in six, sometimes punctures purposely. Would not try such treatment when vitality of patient was low; don't know the proportion saved; saves fully two-thirds of those treated; selects his cases; the majority of indiscriminate cases would be lost.

Dr. Blakesly.—Has never preserved but few nerves; he extirpates them; has not lost one tooth in the past year when nerve has been extirpated and fangs filled; has lost some by capping, perhaps it was owing to their New York climate.

Dr. Blandy.—Always found, when cap touched, the nerve tooth would die. With the foil he forms an arch, or, as he calls it, "chokes the cavity," he then case-hardens the arch thus formed, and fills over it.

Dr. Taft.—Has tried arching and failed; serum will exude from the pulp into cavity under the arch, the final result, the death of the tooth.

Dr. Blandy.—Pathology never tries to save a nerve when inflammation has existed; thinks that if serum is exuded into arch cavity it forms a cicatrix, and finally sub-dentine.

Dr. Taylor.—Can't trace the period when he commenced treating nerves; knows he did not try it much when young. His plan used to be to strike up a cone in gold; nerve would have a chance to expand into the cavity in cone; found this would not do; thought it owing to the confined air, producing pressure, and acting as irritating agent; don't want pressure or vacuum, hence, approves the mode of Dr. Taft; does not as often attempt to save nerves as formerly.

Dr. Ulrey.—Finds most successful practice to be removing the nerve; likes Taft and Taylor's mode of treating nerves; had used camphor and kreosote, and punctured, and had found cases years after with hard dentine where the nerve had been exposed.

Dr. Webster.—Has been universally unsuccessful in capping.

Dr. H. R. Smith.—Has used a great many ways of treating nerves, not all successful; would like to hear of some of the failures; can't see what great good is to result from puncturing the membrane. If few drops only are drawn, as gentlemen say they do, these few drops come from above, and their place is immediately filled by three others, and so on.

Now what good can drawing three drops do? just none at all; puncture, he thinks, will produce inflammation; if he wounds a nerve he applies chloride of zinc, or tincture of myrrh and glycerine; when great inflammation exists, don't try to save nerve; takes it out; does not believe that after nerve is wounded it will assume healthy action; does not use capping, arching, or non-conductors.

### *Filling Teeth.*

Dr. Blakesly.—He uses Watts' crystal gold foil for filling. He uses it because it is absolutely pure; he understood they had a peculiar mode of refining it which others did not possess. Many had experienced difficulty in the use of crystal gold. The reason of this is, sometimes Watts had to leave its manufacture to others, and thus sometimes it was got out nice, and sometimes it was not. He, Dr. B., never did like crystal gold, and he had been urging Watts & Co. a long time before they consented to make foil for him; since they had, he had been able to do a great deal with it; he sometimes uses foil non-adhesive underneath and finishes with adhesive; never could build out before he got Watts' foil or sponge gold; drills pits in the surface of cavity, fills these as separate points, and builds foils on one of these which acts as an anchor; from this he builds to the other points; uses broaches for sinking these pits, as well as for removing decay in the connecting lines of crucial cavities in grinding surface of molar teeth; cuts a leaf of foil into two or three pieces, and rolls these into a delicate rope; cuts this into pieces about as long as broad, with these he fills. If gold is inclined to move, rests another instrument against it; his largest plugger, at the point, fits into No. 27 of Stubb's gauge; usually fills with much smaller points; exhibited the first filling he had put in on the adhesive plan.

All this excellency of filling by building he had reached within one year; could entirely restore the form of teeth to the natural outline. It takes him a great deal of time, but under it he had trebled his prices and doubled his business.

Dr. Taylor.—Thought building out gold to restore the form of the teeth was frequently carried too far for the safety of the filling, and unnecessary for the preservation of the tooth. He had done some of it for glory, but when does it pay? He thought that in back teeth it should only be done when an antagonizing point was very much needed; he had been using adhesive foil for several years, made by James Leslie of our city, (Cincinnati,) and had no need for any thing more adhesive. He alluded to the experiments made a year ago before the association by this gentleman, in which he explained the mode of analyzing foil, and demonstrated the adhesive property of pure gold when in the form of foil. Dr. T. also showed the finger-ring made by A. M. Leslie from scraps of his adhesive foil, without melting or soldering, and which was held together by the adhesive property alone of the gold; he had now worn it constantly for one year, and it was evidently as good as the day he put it on. (Dr. Blakesly asked if he had *seen* it made.) Dr. T. uses blocks a great deal in filling, and so overcomes by rapidity the flow of saliva.

Dr. Hunter.—Took for a case, to describe his mode of filling, a cavity in the centre of a molar tooth; having formed cavity rightly, he divided his foil into different sized pieces, (sometimes using whole sheets,) as the size of the cavity will permit; these he rolls up into balls or pellets; he puts in as large a one as he can get into the cavity, and with a large pointed plugger comes down on it, if in the under jaw, or up on it, if in the upper jaw, and presses it home; picks up another and socks it home, and so on, until he has got the cavity a little more than the thickness of enamel from full; he then takes adhesive foil, and, with a fine pointed instrument, welds it on the condensed surface of the filling, until he fills the cavity perfectly full. This last adhesive solid coat he views as the *enamel ;* the less hard filling underneath as the *dentine*, and considers these relations more in harmony with nature.

Dr. Isaiah Forbes.—Supposed a case: A cavity in the approximal surface of an upper bicuspid tooth, one-third of the crown of the tooth being gone, including as much enamel of the grinding surface. Having formed his cavity properly, he filled such a cavity by using large blocks, the first one frequently being formed of one sheet, softly but regularly folded, and finished with varying sizes, as best suited the case.

Dr. Blakesly.—Would not wish to be understood as advising building teeth out in all cases ; did not generally do it in approximal cavities of bicuspid teeth; alluded to it more to show what could be done with the foil he used, than to recommend it as a general rule. He thought with Dr. Taylor, that it is best in back teeth to fill but little beyond the cavity. He also thought well of cylinders, and had no doubt fillings could be put in faster thus than by the mode he pursued. A year and a half ago, met Dr. Clark, of New Orleans, and he told him how to fill fangs. He (Dr. B.) never uses a tempered instrument in fang; don't fill every fang, because he can't; he does not use wire in filling fangs; uses little points of pure gold, runs these up and breaks them off.

The surface of the gold filling must be kept dry. If it gets wet, he stops and talks and takes a rest; dries the surface of gold with paper, then wets it with chloroform, and wipes again. This restores adhesion. Don't recollect now when he had a cavity overflow ; had filled cavities wet, and saw no difference, but did not preach it.

Dr. Leslie said he would occupy a short time in making a few remarks on materials for filling. First, he would say of crystal gold foil, that if any remark he could make would induce any present to try it, he would cheerfully make it. He did not know that he could say more than that there was no more adhesive foil in the market than it, and he kept it for sale. Of Leslie's foil he had nothing to say here. He had not come for the purpose of talking it up, his purpose was very different. Its reputation he was willing to leave in the hands of those who had used it. There was one claim made for the crystal gold foil, which had been often repeated here to-day, which he wished to notice. It has been claimed for the foil that it was purer than all other foils. This is the claim made by Watts & Co., and repeated by Dr. Blakesly here. Let us look back into the history of this claim of extra purity, and see what it reveals. It will be recollected that when crystal gold was first introduced, a *similar* claim was set up for it. That Dr. Dwinelle, in an article written at that time, and published in the American Journal, undertook to write down foil, and write up crystal gold, and this same claim of "always absolutely the same," as to purity, was a corner-stone in the fabric. Now, we know, that in this the doctor was found sadly mistaken. It was found afterwards that it was *not* "always absolutely the same." A *better* way was afterwards found, and the former make requested to be returned. Of the improved crystal gold, the *same* claim was made as of the first sent out. With either, foil was to stand no chance. The wonders that crystal gold would accomplish, were truly astonishing. But lo! what do we hear?—why, that these clever discoverers have found out that foil is of more service than the much vaunted crystal gold. That they have arrived at this conclusion, they are, perhaps, as much indebted to the speaker as to any one else. It will be remembered that, at the introduction of crystal gold, I took grounds, in the American Journal, in defence of foil, and *first* developed *its adhesive property*, in reviewing Drs. Dwinelle and Taft. What then has become of all these positive claims for crystal gold and its purity?—gone, and experience has added its wisdom. It ought to have taught Watts & Co. to have been more cautious in announcing their crystal gold foil. Already we are told *it* has improved. Dr. Blakesly tells us on this floor that the foil they have made within the last four weeks is more adhesive than any they have before made. I would advise them to learn from their experience in crystal gold, not to be always so "absolutely the same thing," and to remember that other people do not believe that Watts & Co. absorb all chemical knowledge touching gold.

Dr. Watts said, that while residing in Xenia, he was called on by an agent who made this same claim for Watts & Co's mode of refining that Dr. Blakesly had done, and as he had and did yet consider the members of that firm as his personal friends, he requested the agent to say to them, that it was his advice that they cease making this claim, as it was absurd; and in case he had failed to convey that message, he would now request Dr. Blakesly to convey this advice to them as an act of friendship. He had analyzed Watts', Leslie's, and several other makes of foil, and could say of them all, that they might be considered absolutely pure.

It is folly to suppose that chemical knowledge touching gold can be confined to one man.

An agent of A. J. Watts had admitted to him that Dr. Dwinelle had over-acted in the matter, to their injury it had proved.

Dr. Blakesly.—Said Watts & Co., not knowing any thing practically of dentistry, had got Dr. Dwinelle, as he had used much of their gold, to prepare a treatise on it; but they had much regretted that he had taken such strong grounds on the matter. It had been greatly to their injury the manner in which it had been introduced; he had been too hasty in saying and publishing what he did.

Owing to absence, we find our notes of other discussions too meagre to be of interest. The attendance, including those not active members, was good, and the impression was that there had been a good meeting. The next meeting will be held in February, 1859. The officers elect are:

*President*—W. R. Webster.

*Vice-President*—F. W. Keely.

*Corresponding Secretary*—J. Taft.

*Recording Secretary*—G. Watt.

*Treasurer*—C. Bonsall.

*Executive Committee*—J. P. Ulrey, C. N. Woodward, H. R. Smith.

*Committee on Membership*—E. Osmond, A. G. Stipher, J. Richardson.

*Committee on Dental Progress*—J. Taylor, H. A. Smith, J. Taft. [*Am. Dent. Review.*

